# Consequential Impact of Particulate Matter Linked Inter-Fibrillar Mitochondrial Dysfunction in Rat Myocardium Subjected to Ischemia Reperfusion Injury

**DOI:** 10.3390/biology11121811

**Published:** 2022-12-13

**Authors:** Bhavana Sivakumar, Abdullah F. AlAsmari, Nemat Ali, Mohammad Waseem, Gino A. Kurian

**Affiliations:** 1 Vascular Biology Laboratory, School of Chemical and Biotechnology, SASTRA Deemed University, Thanjavur 613401, Tamil Nadu, India; 2 Department of Pharmacology and Toxicology, College of Pharmacy, King Saud University, Riyadh 11451, Saudi Arabia; 3 Department of Pharmaceutical Sciences, School of Pharmacy, University of Maryland Eastern Shore, Princess Anne, MD 21853, USA

**Keywords:** particulate matter, inter-fibrillar mitochondria, diesel exhaust, ischemia reperfusion injury, cardiomyocytes

## Abstract

**Simple Summary:**

Inhalation of particulate matter (PM_2.5_) is known to cause cardiac effects and exacerbate any pre-existing cardiac diseases. However, the severity of toxicity depends on how much PM_2.5_ has reached the blood circulation and then finally the heart. The consequential impact of PM_2.5_ on the heart depends on its potential to alter cardiac oxidative stress and mitochondrial function that in turn controls the contractile function of the heart. In fact, this effect is partly concentration dependent. In the present study, we demonstrated that blood borne PM_2.5_ can inflict cardiac injury and compromise physiological response. Moreover, the results from our study show that cardiac tolerance to resist ischemia reperfusion injury (IR) is low in PM_2.5_ administered rat hearts. Cellular level analysis of the data suggests that PM_2.5_ gets deposited in the mitochondria which in turn increases the oxidative stress by disturbing redox couple and inducing mitochondrial dysfunction. In fact, the higher pathological changes occur with the direct entry of PM into the myocardium.

**Abstract:**

A previous study has reported that exposure to PM_2.5_ from diesel exhaust (diesel particulate matter (DPM)) for 21 days can deteriorate the cardiac recovery from myocardial ischemia reperfusion injury (IR), where the latter is facilitated by the efficiency of mitochondrial subpopulations. Many investigators have demonstrated that IR impact on cardiac mitochondrial subpopulations is distinct. In the present study, we decipher the role of PM_2.5_ on IR associated mitochondrial dysfunction at the subpopulation level by administrating PM_2.5_ directly to isolated female rat hearts via KH buffer. Our results demonstrated that PM_2.5_ administered heart (PM_C) severely deteriorated ETC enzyme activity (NQR, SQR, QCR, and COX) and ATP level in both IFM and SSM from the normal control. Comparatively, the declined activity was prominent in IFM fraction. Moreover, in the presence of IR (PM_IR), mitochondrial oxidative stress was higher in both subpopulations from the normal, where the IFM fraction of mitochondria experienced elevated oxidative stress than SSM. Furthermore, we assessed the in vitro protein translation capacity of IFM and SSM and found a declined ability in both subpopulations where the inability of IFM was significant in both PM_C and PM_IR groups. In support of these results, the expression of mitochondrial genes involved in fission, fusion, and mitophagy events along with the DNA maintenance genes such as GUF1, LRPPRC, and HSD17-b10 were significantly altered from the control. Based on the above results, we conclude that PM_2.5_ administration to the heart inflicted mitochondrial damage especially to the IFM fraction, that not only deteriorated the cardiac physiology but also reduced its ability to resist IR injury.

## 1. Introduction

Elevated cardiovascular diseases associated with air pollution exposure, which is reported in the recent times, is linked to the toxicity induced by particulate matter (PM) present in the pollutant [1]. Recent publications have reported that PM_2.5_ (PM < 2.5 μm in size) accounted for nearly 200,000 premature deaths annually [2]. Inhaled PM contains a fraction of inorganic/metals and organic/polycyclic aromatic hydrocarbons that are associated with oxidative stress and inflammation [3]. The pathological impact of respired PM_2.5_ involves a series of cellular events in the vasculature that includes endothelial dysfunction that act as the initial trigger for the development of cardiovascular diseases (CVD) [4]. In addition to inflammation, PM_2.5_ can also promote oxidative stress, metabolic dysfunction, alteration of lipid metabolism, and autonomic dysfunction. All these mediators in turn can adversely affect the cardiac contractile function and heart rate [5].

Mitochondria play a key role in regulating the cardiac excitation and contraction coupling in myofibrils by supplying energy via oxidative phosphorylation (OXPHOS) [6]. Moreover, they regulate homeostasis of Ca^2+^ and control different metabolic pathways to facilitate normal cardiac function. Previous studies by our group demonstrated that PM_2.5_ exposure for 21 days can perturb mitochondria and can act as a source of free radical release that can induce oxidative stress [7,8]. Basal level mitochondrial alterations in PM_2.5_ exposed rat heart exhibited higher cardiac injury during ischemia reperfusion injury (IR).

Myocardial tissue contains a discrete pool of mitochondria that resides below the sarcoplasmic reticulum, termed subsarcolemmal mitochondria (SSM), and those residing between the contractile units of myofibril, are termed as interfibrillar mitochondria (IFM) [9]. Early studies have hypothesized that IFM supplies energy for the contractile unit as they reside in the myofibrils [10]. On the other hand, SSM can also produce ATP which are predominantly used for the active transport of electrolytes and metabolites [11]. Besides mitochondria being the metabolic hub of many biochemical pathways, SSM fraction of mitochondria is predominantly involved in cellular homeostatic function by regulating different metabolic pathways.

PM_2.5_ induced cardiac abnormalities are primarily associated with cardiac physiology and contractility [12]. A recent study has reported that ambient PM induced cardiotoxicity is associated with disrupted PPAR/PGC alpha signaling and mitochondrial damage [13]. In addition, PM_2.5_ can inflict mitochondrial damage by altering the redox imbalance in the tissue [14]. Being present in the myofibrils, we suspect that the PM_2.5_ induced damage is severe in the IFM fraction. This also raised the question whether PM_2.5_ linked mitochondrial damage is more specific to IFM fraction of mitochondria, due to its spatial location that may be responsible for PM_2.5_ associated contractile decline. Many studies have shown that deteriorated mitochondrial function can reduce the cardiac tolerance to withstand ischemia reperfusion injury (IR) [15]. Early reports from our lab emphasize IFM damage associated with IR which in turn adversely affects the cardiac contractility [16]. However, SSM appears to be more vulnerable to ischemic injury and mitochondrial Ca^2+^ overload when compared to IFM [17]. But how these subpopulations behave in presence of PM_2.5_ induced stress conditions is unknown. In the present study, we explore the adverse effect of PM_2.5_ on the functioning of mitochondrial subpopulations and how it affects the cardiac recovery from IR. These findings support the emergent view that myocardium possesses distinct mitochondrial subpopulations that are inherently unique and displays distinct adaptations in response to energy stress.

## 2. Materials and Methods

### 2.1. Animals

All procedures for animal studies were approved by Institutional Animal Ethics Committee (IAEC), SASTRA University, Thanjavur, India (CPCSEA Approval No./SASTRA/IAEC/RPP/709). This study was conducted in accordance with the CPCSEA (Committee for the Purpose of Control and Supervision of Experiments on Animals) guidelines. All rats were well maintained in polycarbonate cages and were maintained at a temperature and humidity of 25 ± 2 °C and 65 ± 2%. Feed and water were provided ad libitum.

### 2.2. Animal Experimental Groups

Eight to twelve weeks old wistar rats weighing 200–250 g were divided into 4 groups having 6 animals each: (1) Normal (N), (2) Ischemia reperfusion (IR), (3) PM_2.5_ control (PM_C), and (4) PM_2.5_ IR (PM_IR). The rats were anaesthetized with 60 mg/kg1 i.p. of sodium thiopentone, after which the hearts were excised and mounted on Langendorff isolated rat heart apparatus (AD Instruments Pvt. Ltd, Sydney, Australia) and perfused with Krebs–Henseleit (KH) buffer at a constant pressure as per the requirement of the treatment groups [18]. The apparatus was maintained at a constant temperature of 37 °C throughout the course of the experiment (stabilization, ischemia, and reperfusion) using the thermostat. The isolated rat hearts from the normal control groups (N) were perfused continuously with KH buffer for 120 min. The hearts in the IR group were stabilized for 30 min, followed by 30 min of ischemia and 60 min of reperfusion. The hearts in the PM_C group were treated with 100 µg/mL of DPM (SRM_2975, National institute of standard and technology) via KH buffer for 20 min.

According to air quality index (AQI), the concentration which we used belonged to the unhealthy range. The bioavailability of PM_2.5_ in an ex vivo isolated rat heart model is relatively higher than that of whole-body exposure as PM_2.5_ was directly perfused to the heart and hence we fixed the concentration as 100 µg/mL [19,20]. According to Li et al. 2019, an 8 h exposure to PM_2.5_ leads to a deposition of 15.6 μg/kg body weight. For a human being weighing 70 kg, when exposed to PM for 8 h, they are measured to have 15.6 × 70 = 1092 μg ≈ 1 mg of pollutant in the blood. The toxic impact of pollutant will be precipitated once it gets deposited in the specific organ at a considerable range and starts producing secondary effect. Over the period of exposure, the heart may experience a higher concentration of PM_2.5,_ especially when the subject is predisposed with morbidities that incur cardiomyopathy.

In the PM_IR group, post-DPM treatment, the hearts underwent the IR protocol. All hearts were stored at −80 °C for analysis until use. The metal composition of SRM_2975 is as follows: Ca, Mg, Al, S, K, Fe, Zn, Cu, Cr, Pb, Ba, and Ni [21] (https://www-s.nist.gov/srmors/certificates/2975.pdf accessed on 4 November 2022).

### 2.3. Assessment of Cardiac Hemodynamics and Injury Parameters

Cardiac recovery was assessed by measuring the hemodynamic changes using Labchart Pro 8 and Power Lab Data Acquisition System (AD Instruments, AD Instruments Pvt. Ltd, Sydney, Australia). The heart rate, left ventricular developed pressure (LVDP), left ventricular end-diastolic pressure (LVEDP), and rate pressure product (RPP) were determined. Creatinine kinase myocardial band (CKMB) level was measured using the CKMB kit-14410005 from Agappae (Kochi, India). Triphenyl tetrazolium chloride (TTC) staining and H&E staining were carried out as previously described. Apoptosis was analysed using TUNEL staining (Takara Bio Inc., San Jose, CA, USA) as per the manufacturer’s instructions and triphenyl tetrazolium chloride (TTC) staining was carried out for injury assessment at the structural level. The gene expression of caspases-3,7,9 and PARP were studied using real-time PCR technique as previously mentioned [18].

### 2.4. Assessment of Mitochondrial Electron Transport Chain Enzyme Activities and Oxidative Stress

Mitochondrial subpopulations, namely IFM and SSM, were isolated via differential centrifugation techniques as previously described with slight modifications [22]. Briefly, after tissue homogenization, and subsequent centrifugation at 600 g for 10 min at 4 °C, the pellet obtained was further treated with trypsin for IFM isolation, whereas the supernatant was utilized for SSM isolation. For IFM isolation, the homogenate pellet obtained was incubated in trypsin (0.5 mg/g) for the tissue digestion that enables the release of IFM. The pellet was suspended in the isolation buffer and centrifuged at 600 g for 10 min at 4 °C. The supernatant was then collected and centrifuged at 12,000× *g* for 10 min at 4 °C to pellet out the IFM fraction. A similar protocol was adopted for the supernatant obtained in the first centrifugation step for SSM isolation as well. The pelleted mitochondrial subpopulations were suspended in storage buffer ((in mM) 25 sucrose, 75 sorbitol, 10 Tris-HCl, 100 KCl, 10 K2HPO4, 5 MgCl2, and 0.05 EDTA) and stored at 4 °C for biochemical analysis.

A Synergy H1 multimode reader was used to measure the activity of the mitochondrial ETC enzymes NADH-oxidoreductase (NQR), succinate decylubiquinone DCPIP reductase (SQR), ubiquinol cytochrome c reductase (QCR), and cytochrome c oxidase (COX) (BioTek, USA). Decylubiquinone was used to record the oxidation of NADH by complex I, and the decrease in absorbance was measured at 340 nm using a molar absorption coefficient of ε = 6.2 mM^−1^ cm^−1^. At 600 nm, complex II activity was determined by how quickly 2,6-dichlorophenolindophenol (DCIP) was reduced (ε = 21 mM^−1^ cm^−1^). The rate of cytochrome c reduction at 550 nm (ε = 18.5 mM^−1^ cm^−1^) was used to measure the activity of cytochrome c reductase, also known as complex III activity. By measuring the rate of cytochrome c oxidation at 550 nm (ε = 19.6 mM^−1^ cm^−1^), complex IV activity was identified. In the isolated mitochondria of all the experimental groups, ATP estimate was carried out using the ATPlite (Perkin Elmer). The lysis buffer was added to 50 µL of mitochondria and shaken for 5 to 10 min. The mitochondria were then incubated in the dark for 20 min after the addition of the substrate buffer, and luminescence was then detected [8].

Antioxidant levels were measured as follows: Through the addition of 12 mM pyrogallol solution to 50 µg of mitochondria in Tris-HCl buffer with 1 mM Na2EDTA, the superoxide dismutase (SOD) activity was determined. At 420 nm, the absorbance was calculated. Units per milligramme of protein were used to measure SOD activity. In millimoles of H_2_O_2_ decomposed per minute per milligram of protein, catalase activity was measured. Briefly, 50 µg of mitochondria was placed in a 50 mM potassium phosphate buffer along with 20 mM of H_2_O_2_ as a substrate. At a wavelength of 240 nm, the reaction was read for 15 min at intervals of 1 min. The oxidation of GSH by 2-nitrobenzoic acid (DTNB) to produce the yellow derivative 5′-thio-2-nitrobenzoic acid (TNB) at 412 nm allowed researchers to identify GSH. In the presence of NADPH, glutathione reductase can convert the GSSG produced back to GSH.

### 2.5. Gene Expression Studies

According to the manufacturer’s instructions, the total RNA was extracted using the TRIzol reagent (Thermo Fisher Scientific, Waltham, MA, USA). The reverse transcription was carried out using Thermo Fisher Scientific’s Verso cDNA synthesis kit. Thermo Fisher Scientific’s DyNAmo Flash SYBR Green qPCR kit was used to perform real-time PCR on a PCR system (ABI 7500, Applied Biosystems, Foster City, CA, USA). The following amplification conditions were used: initial denaturation at 95 °C for 2 min, followed by 35 cycles at 95 °C for 30 s and 60 °C for 30 s.

Following the manufacturer’s instructions, the phenol-chloroform-isoamyl alcohol technique was used to isolate the DNA. Using a Nanodrop ND-1000 spectrophotometer, the concentration of total DNA was determined. As previously mentioned, the TRIzol reagent (15596026, Thermo Scientific) was used for the mRNA extraction. The DNA isolation for mitochondrial copy number estimation was performed using phenol-chloroform-iso-amyl alcohol method according to the manufacturer’s instructions. The concentration of total DNA was measured using a Nanodrop ND-1000 spectrophotometer. The gene expression was assessed in triplicate, and the qPCR results were validated by three separate assays. The Applied Biosystems Step-One Plus PCR system was used to carry out the PCR. In order to examine the data, differences in fold-changes in gene expression were compared using the ΔΔCt method [18].

The primer details are given in Table 1.

### 2.6. MtDNA Copy Number Estimation

mtDNA copy number was determined using a real-time quantitative polymerase chain reaction (qPCR). Using DNA as a source material, the ratio of the relative expression of the MT-RNR2 gene and beta actin gene expression was used to compute the number of mitochondrial DNA copies. The cycle threshold (Ct) value was determined from the amplification curve for each target [23].

### 2.7. Analysis of In Vitro Protein Translation Capacity of Mitochondria

In vitro protein synthesis was performed by suspending the SSM and IFM fraction of mitochondria from different groups in MAITE buffer (in mM: 25.00 sucrose, 75.00 Sorbitol, 10.00 KCl, 0.05 Tris-HCl, and 10.00 K2HPO4, pH = 7.4) containing 10 mM glutamate, 2.5 mM malate, 1 mM ADP, and 1 mg/mL of fatty acid-free BSA. A total of 100 μg/mL emetine, 100 μg/mL cycloheximide, and 10 μM of the 20 L-amino acids were added to the MAITE medium to initiate translation. The mitochondria were incubated at room temperature for 45 min. Translation process was halted at the end of 45 min when the mitochondria were pelleted at 12,000× *g* for 10 min at 4 °C. Sodium dodecyl sulfate–polyacrylamide gel electrophoresis (SDS PAGE) (with 12% resolving gel) was performed to visualise the protein bands. Resultant gels were incubated in Coomassie blue stain for 4 h and was further destained in destaining solution for 2 h. These were imaged using Quantity One software (Bio-Rad, Irvine, CA, USA). The data obtained were used to calculate the protein fold increase after in vitro protein translation [24].

### 2.8. Inductively Coupled Mass Spectrophotometry Analysis

Mitochondria isolated from the respective groups were subjected to ICPMS analysis (ELAN 6000, PerkinElmer-Sciex, Waltham, MA, USA) to quantify the metals found in DPM. Analytical curves in the range of 50–100 µg/L were prepared for calculation. The values are presented in PPM.

### 2.9. Statistical Analysis

Data are represented as the mean ± SD. The significance level between the groups was assessed with one-way ANOVA test followed by Dunnett’s test and post-hoc analysis using Graph Pad Prism 7.0 software (GraphPad Software Inc., San Diego, CA, USA).

## 3. Results

### 3.1. Administration of PM_2.5_ Aggravated Cardiac Injury and Compromised Cardiac Hemodynamics in Isolated Rat Myocardium

**Figure 1:**
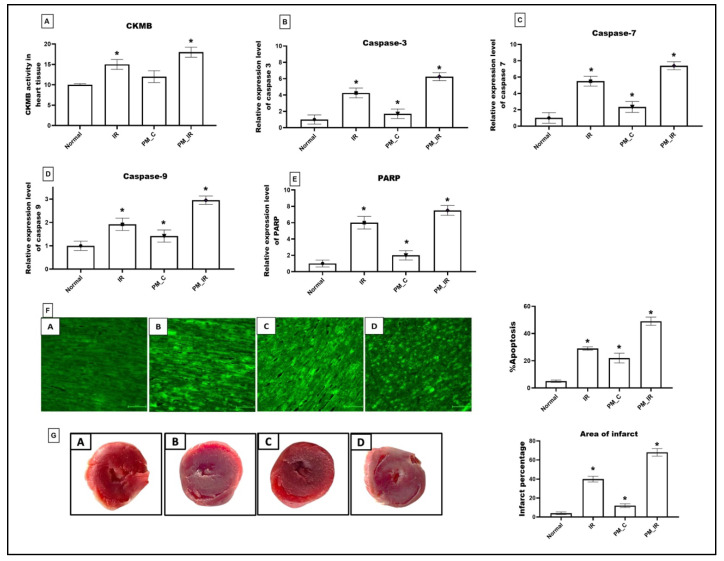
Cardiac injury assessment. (**A**) CKMB level in myocardium. (**B**) Relative gene expression levels of caspase 3. (**C**) Relative gene expression levels of caspase 7. (**D**) Relative gene expression levels of caspase 9. (**E**) Relative gene expression levels of PARP. (**F**) TUNEL staining (40× magnification) (* *p* < 0.05 vs. Normal) (**G**) TTC stained myocardium and calculation of area of infarct using ImageJ software version 1.53t (J software, Rockville, MD, USA). Values are mean ± SD, (*n =* 6 per group) [Groups: (A) Normal, (B) IR, (C) PM_C, (D) PM_IR].

**Figure 2:**
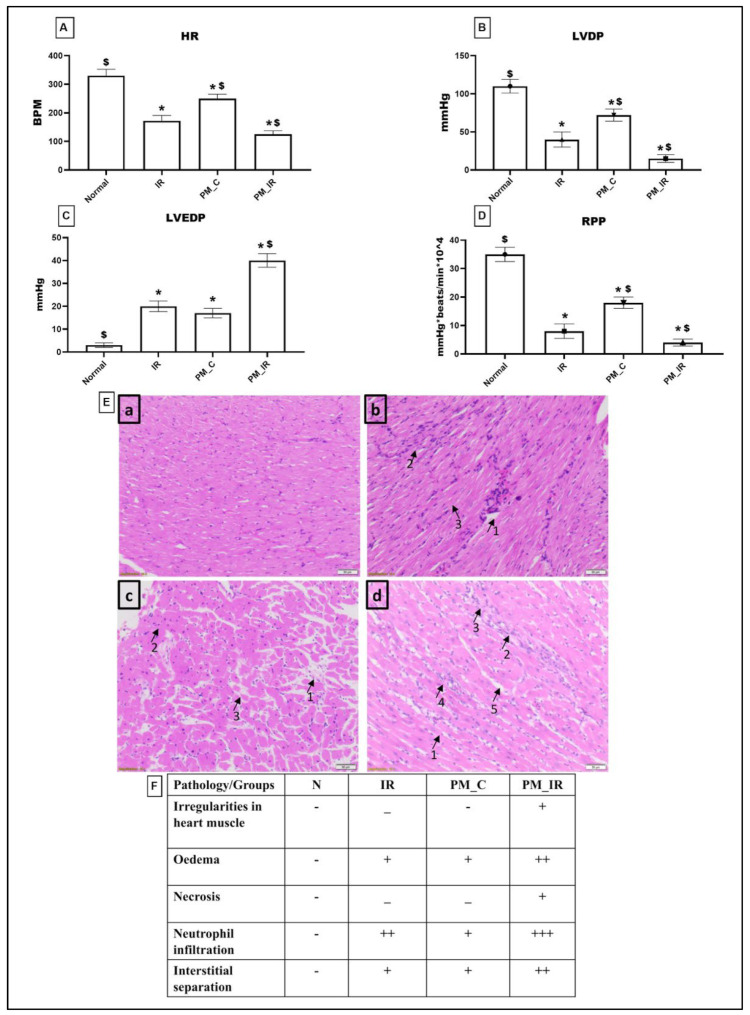
Analysis of cardiac hemodynamic parameters. (**A**) Heart rate (HR). (**B**) Left ventricular developed pressure (LVDP). (**C**) Left ventricular end-diastolic pressure (LVEDP). (**D**) Rate pressure product (RPP). (* *p* < 0.05 vs. normal, ^$^ *p* < 0.05 vs. IR control). Values are mean ± SD (*n =* 6 per group). (**E**) H&E staining of rat myocardium form different groups where (a): Normal, (b): IR, (c): PM_C, (d): PM_IR. (The pathologies are as follows: (b): 1—odema, 2—neutrophil infiltration, 3—interstitial separation. (c): 1—odema, 2—neutrophil infiltration, 3—interstitial separation. (d): 1—irregularities in heart muscle, 2—odema, 3—necrosis, 4—neutrophil infiltration, 5—interstitial separation) (10× magnification with haematoxylin and eosin stain; scale bar = 100 μm). (**F**) Histopathological scoring table. [Groups: (1) Normal, (2) IR, (3) PM_C, (4) PM_IR].

Figure 1 shows the molecular injury parameters that get altered during PM_2.5_ administration. In the normal perfused heart after PM_2.5_ administration via KH buffer (PM_C), the expression of caspase 3,7,9 and PARP gene was significantly elevated (41%, 57%, 29%, and 50%), which was in agreement with the elevated apoptotic injury measured via TUNEL positive cells from the normal control. Furthermore, we identified oedema, neutrophil infiltration, and interstitial separation in PM_2.5_ perfused rat heart. However, CKMB elevation was insignificant for the same. Hemodynamic indices were measured by using lab chart software. According to Figure 2, HR, LVDP, and RPP were significantly decreased (24%, 34%, and 48%) in PM_2.5_ treated rat heart from the normal control. Similarly, in PM_IR, we noted an upregulation of apoptotic genes and PARP by 84%, 86%, 66%, and 86%, respectively, from the normal control. Additionally, the CKMB levels increased to 44% (vs. the normal control). The hemodynamics indices also further declined to 62%, 86%, and 88% from the normal control group. The diminished hemodynamic indices were supported with increased pathological changes (oedema, irregularities in heart muscle, mild necrosis and neutrophil infiltration, and interstitial separation) in the H&E-stained myocardium of the PM_IR group and increased infarct size (Figure 2). Comparison of the PM_C with the PM_IR group displayed a significant increase in the expression of caspases and PARP expression along with the CKMB levels.

### 3.2. Administration of PM_2.5_ Altered the Mitochondrial Dynamics and Increased Mitophagy in Ischemia Reperfused Myocardium

**Figure 3:**
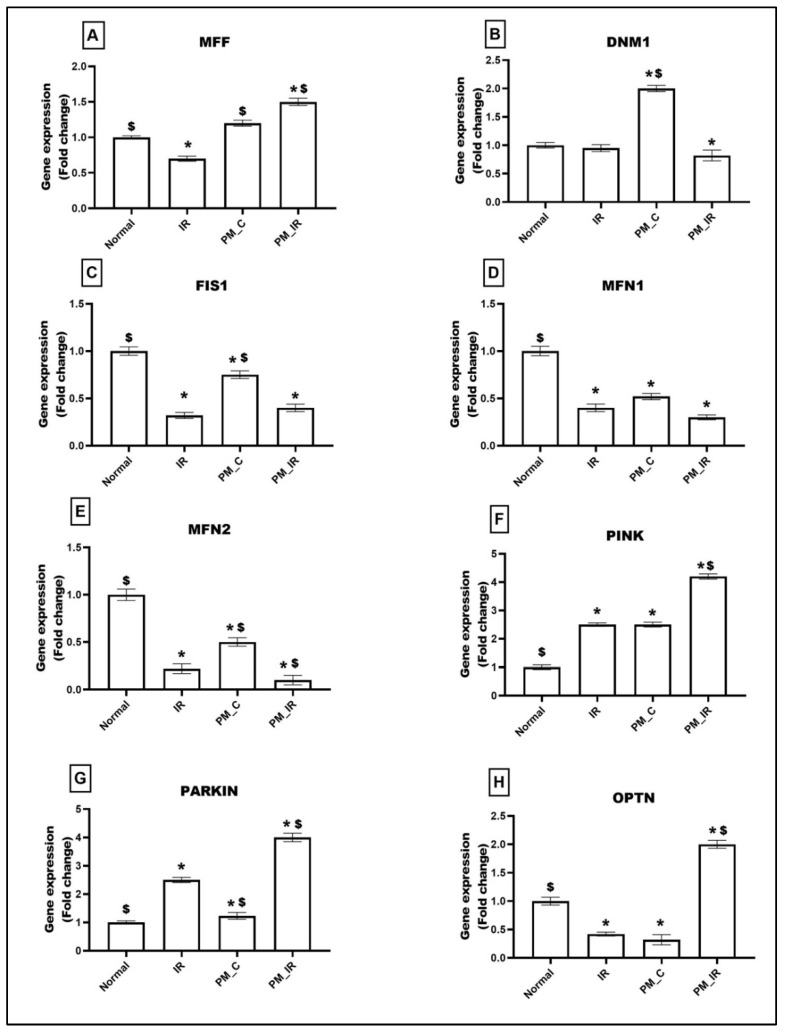
mRNA expression changes of (**A**) MFF, (**B**) DNM1, (**C**) FIS1, (**D**) MFN1, (**E**) MFN2, (**F**) PINK, (**G**) PARKIN, and (**H**) OPTN in myocardium. Values are mean ± SD. Significant difference from control group is indicated by asterisk (* *p* < 0.05, ^$^ *p* < 0.05 vs. IR control). [Groups: (1) Normal, (2) IR, (3) PM_C, (4) PM_IR].

Mitochondrial dynamics and mitophagy events play a significant role in determining the quality of mitochondria. Figure 3 shows the alterations in the expression of genes linked to mitochondrial fission (MFF, DNM1, FIS1), fusion (MFN1, MFN2), and mitophagy (PINK, PARKIN, OPTN) in different experimental groups. PM_2.5_ administered control rat heart (PM_C) displayed an upregulation of DNM1 (50%), PINK (23%), and PARKIN (23%) genes and downregulation of MFN1 (48%), MFN2 (50%), FIS1 (20%), and OPTN genes (68%) (vs. the normal control). Furthermore, in the presence of IR, MFF showed an increased expression of 50% and the expression of DNM1 and FIS1 reduced and decreased by 18% and 60%, respectively, (vs. the normal control). MFN1 and 2 showed a significant downregulation of 70% and 90%, respectively, vs. the normal control. The PM_IR group also displayed increased expression of mitophagy genes (PINK: 76%, OPTN: 50%, and 75% declined expression of PARKIN) than the normal control. Comparison of the PM_IR group with the IR control showed an upregulation of MFF, PINK, PARKIN, and OPTN and a downregulation of DNM1, MFN1, and MFN2.

### 3.3. Administration of PM_2.5_ Altered the Mitochondrial Bioenergetics Function and Elevated Oxidative Stress in the IFM Fraction of Ischemia Reperfused Myocardium

**Figure 4:**
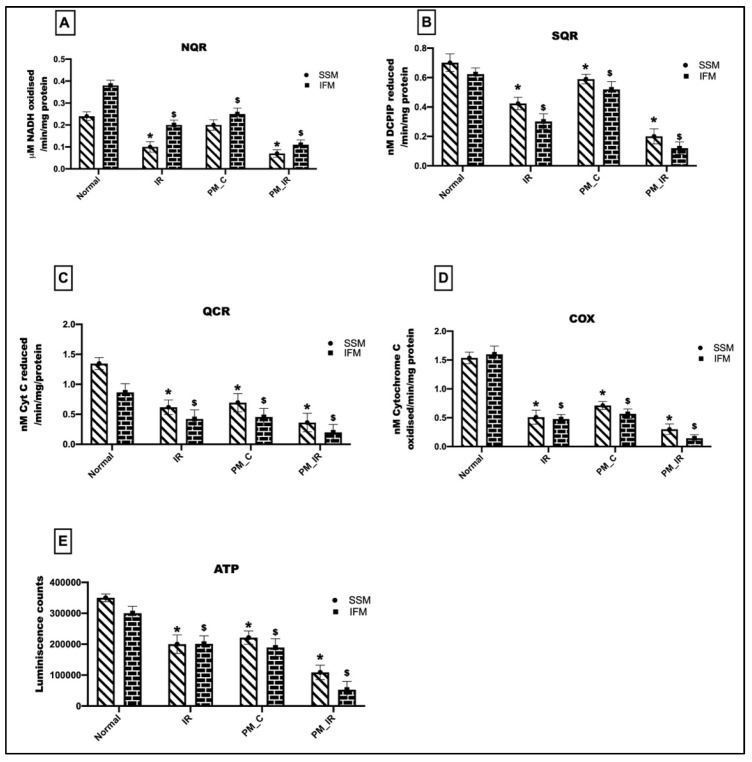
Assessment of mitochondrial ETC complex activities: (**A**) NQR, (**B**) SQR, (**C**) QCR, (**D**) COX, (**E**) ATP assessment. (* *p* < 0.05 vs. normal of SSM, ^$^ *p* < 0.05 vs. normal of IFM). Values are mean ± SD. (*n =* 6 per group). [Groups: (1) Normal, (2) IR, (3) PM_C, (4) PM_IR].

Figure 4 gives the bioenergetic changes in mitochondria with respect to IR, where the hearts were treated with and without PM_2.5_. We observed a 34%, 16%, 48%, and 65% decline in the NQR, SQR, QCR, and COX activities in the IFM fraction of the PM_C group. In the mitochondrial SSM fraction, there was a 17%, 16%, 48%, and 53% decline in the NQR, SQR, QCR, and COX activities of the isolated mitochondria. This was supported by a decline in ATP levels in the IFM fraction of the PM_C group. However, in the PM_IR rat hearts, the declined ETC activities were further deteriorated in both the subpopulations. NQR, SQR, QCR, and COX activities were decreased by 71%, 80%, 78%, and 91%, respectively, in IFM and 70%, 72%, 73%, and 80% in SSM from the control heart. But when compared with the IR control, there was a significant decline in all of the four complex activities in both IFM and SSM fractions, with the highest decline in the IFM mitochondrial fraction. A similar trend was observed in the ATP levels as well.

**Figure 5:**
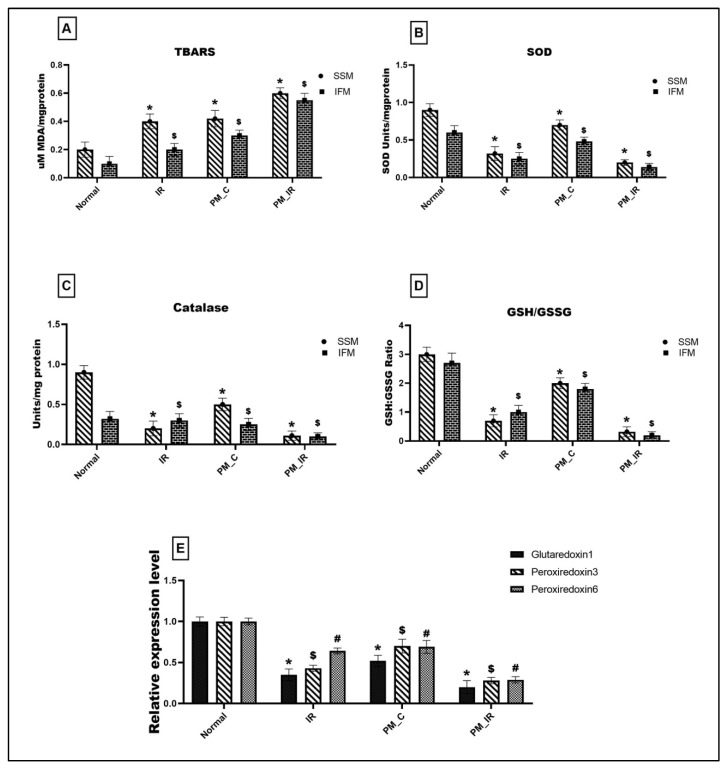
Assessment of mitochondrial oxidative stress. (**A**) Lipid peroxidation: TBARS level, (**B**) SOD, (**C**) catalase, (**D**) GSH:GSSG ratio, (* *p* < 0.05 vs. normal of SSM, ^$^ *p* < 0.05 vs. normal of IFM). (**E**) Gene level expression of glutaredoxin 1, peroxiredoxin 3, and peroxiredoxin 6 where *^,$,#^ *p* < 0.05 vs. Normal, where * corresponds to the normal of glutaredoxin 1, ^$^ to the normal of peroxiredoxin 3, ^#^ to the normal of peroxiredoxin 6, respectively. Values are mean ± SD (*n =* 6 per group). [Groups: (1) Normal, (2) IR, (3) PM_C, (4) PM_IR].

Figure 5A gives the lipid peroxidation level and antioxidant enzyme activities in different experimental groups. PM_C rat mitochondria displayed a significant elevation in the lipid peroxidation level measured via TBARS (66% in the mitochondrial IFM fraction and 52% in the SSM) and oxidative stress (33% in the IFM and SSM fraction) (Figure 5D) from the normal control. The above results were supported by a significant decline in the antioxidant enzyme activities such as SOD (20%) and catalase (28%) in the mitochondria where IFM fraction exhibited a prominent decline. Furthermore, with IR induction, the lipid peroxidation level showed a further upregulation of 66% in the SSM fraction and 81% in the IFM fraction, and antioxidant enzyme also declined to 89% in SSM and 92% in IFM, respectively. The SOD and catalase levels also displayed a similar trend with the IFM population exhibiting severe oxidative stress. Comparing the PM_C with the PM_IR group, the lipid peroxidation increased by 30% in SSM fraction and 46% in IFM fraction, respectively, and the oxidative stress markers showed a significant decline in the IFM fraction as reported previously (GSH/GSSG: 84% in SSM and 88% in IFM). Mitochondrial antioxidant genes such as glutaredoxin 1, peroxiredoxin 3, and peroxiredoxin 6 were measured for their expression levels and the results are given in Figure 5E. Accordingly, PM_C rat mitochondria showed 48%, 30%, and 31% declined expression of glutaredoxin 1, peroxiredoxin 3, and peroxiredoxin 6 from the control. However, in PM_IR rat heart, glutaredoxin 1, peroxiredoxin 3, and peroxiredoxin 6 were downregulated by 43%, 35%, and 58% from the IR control and 80%, 72%, and 71% from the control.

### 3.4. Administration of PM_2.5_ Altered the Expression of Mitochondrial Regulatory Genes and Reduced the Mitochondrial DNA Copy Number in Ischemia Reperfused Myocardium

**Figure 6:**
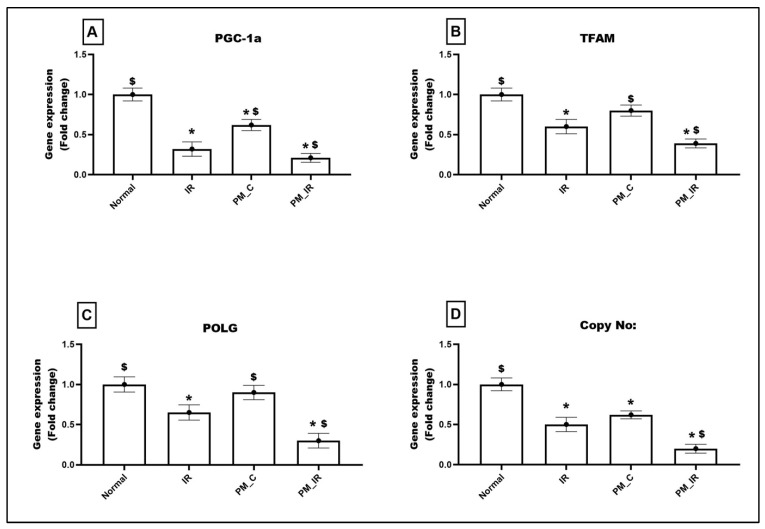
mRNA expression changes of (**A**) PGC1-a, (**B**) TFAM, (**C**) POLG, and (**D**) DNA copy number in the myocardium. Values are mean ± SD. Significant difference from control group is indicated by asterisk (* *p* < 0.05, ^$^ *p* < 0.05 vs. IR control). [Groups: (1) Normal, (2) IR, (3) PM_C, (4) PM_IR].

PM_2.5_ administration declined the expression of mitochondrial regulatory genes such as PGC1-a (38%) and regulatory genes such as TFAM and POLG by 20% and 10%, respectively, from the normal control (Figure 6). With IR induction, the expression of these genes was further downregulated by 79%, 61%, and 70%, respectively, (vs. the normal control). This was supported by low mtDNA copy number in the PM_C group (38%), which was further declined to 80% in the presence of IR (Figure 6). Even when compared with IR, PM_IR hearts exhibited prominent decline in PGC1-a, TFAM, POLG, and mtDNA copy number.

### 3.5. Administration of PM_2.5_ Altered the Expression of Mitochondrial DNA Maintenance Genes in Ischemia Reperfused Myocardium

**Figure 7:**
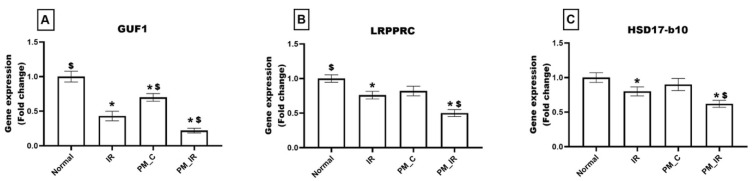
mRNA expression changes of (**A**) GUF1, (**B**) LRPPRC, and (**C**) HSD17-b10 in the myocardium. Values are mean ± SD. Significant difference from control group is indicated by asterisk (* *p* < 0.05, ^$^ *p* < 0.05 vs. IR control). [Groups: (1) Normal, (2) IR, (3) PM_C, (4) PM_IR].

As observed in Figure 7, the PM_C group displayed a significant decline in the expression of GUF1 (30%) and an insignificant reduction in the expression levels of LRPPRC (18%) and HSD17-b10 (10%), respectively, from the normal control. Furthermore, in the presence of IR, PM_2.5_ treated rat hearts displayed significant downregulation of GUF1, LRPPR, and HSD17-b10 by 78%, 50%, and 38%, respectively, (vs. the normal control). Furthermore, from PM_C heart, PM_IR rat hearts exhibited 69%, 39%, and 31% decline in the percentage of the gene expression, respectively.

### 3.6. Administration of PM_2.5_ Decreased the In Vitro Protein Translation Capacity in the IFM Fraction of Ischemia Reperfused Myocardium

**Figure 8:**
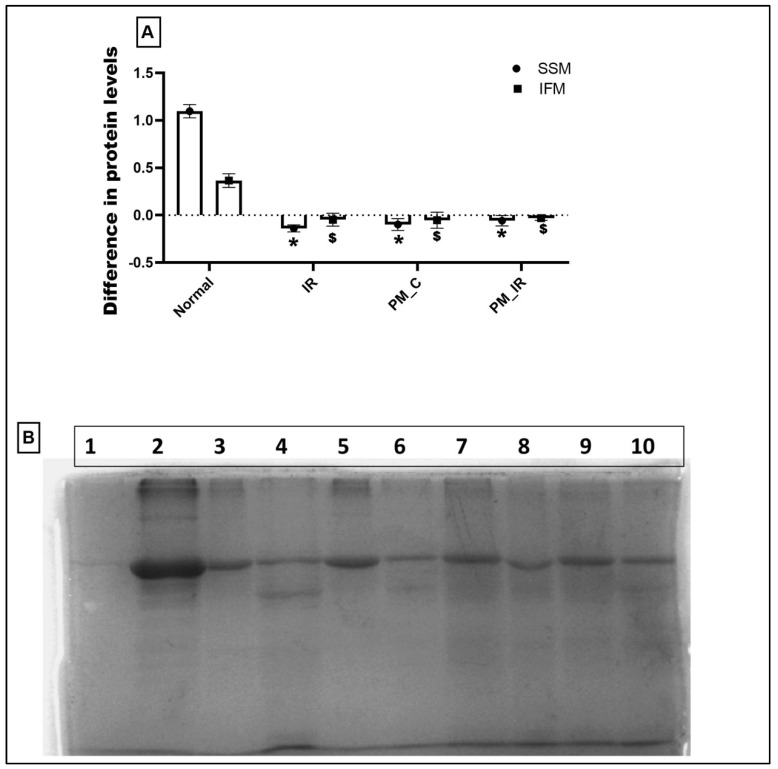
Protein translation ability of mitochondria isolated from the experimental groups. Lanes: 1: Negative control, 2: Positive control, 3–6: IFM fraction of N, IR, PM_C, and PM_IR groups, 7–10: SSM fraction of N, IR, PM_C, and PM_IR groups. (**A**) The difference in protein levels between the different experimental groups. (**B**) Protein translation ability of mitochondria isolated from the experimental groups. Lanes: 1: Negative control, 2: Positive control, 3–6: IFM fraction of N, IR, PM_C, and PM_IR groups, 7–10: SSM fraction of N, IR, PM_C, and PM_IR groups. (* *p* < 0.05 vs. normal of SSM, ^$^ *p* < 0.05 vs. normal of IFM) (Supplementary Materials).

Figure 8 shows the in vitro protein translation capacity of isolated mitochondria obtained from different experimental groups. Compared to the normal cardiac mitochondria, mitochondria from the PM_C group did not show signs of protein translation, rather degradation of protein was noted in both the mitochondrial subpopulations, namely IFM and SSM. In fact, this decline was severe in the IFM fraction, which is supported by SDS PAGE data. According to Figure 8B, the band intensity of IFM was lower in the PM_C and PM_IR groups than the SSM fraction, indicating the possibility of severe damage in the IFM fraction.

### 3.7. ICPMS Data Reveals the Deposition of Metal Components from PM_2.5_ in the Mitochondria of Hearts Administered with PM_2.5_

Table 2 displays the level of metal components from DPM in cardiac mitochondria. We observed an increase in the accumulation of Na, K, Ca, Mn, Fe, and Cu in the mitochondria of hearts administered PM_2.5_. The presence of additional metals such as B and Pb was also observed.

## 4. Discussion

Early studies have shown that PM_2.5_ from DPM enhances the sensitivity of the heart to injury associated with infarction [8,16] and ischemia reperfusion [7,25,26]. In both the cardiac anomalies, mitochondrial dysfunction was reported to be the prime causative reason. However, cardiac mitochondria comprise of interfibrillar and subsarcolemmal mitochondria (IFM and SSM), where the former functions to provide energy to the myofibrils for contraction, while SSM mainly regulates the cardiac metabolic homeostasis [17]. Contractile dysfunction was reported to be associated with PM_2.5_ toxicity in the heart [27]. However, the existing literature does not provide sufficient evidence for the direct role of PM_2.5_ on the mitochondrial subpopulations and the subsequent impact on cardiac physiology and tolerance to resist IR. The major findings of the present study showed that PM_2.5_ deteriorated the bioenergetics function of IFM and that was in turn manifested into decreased cardiac contractility of PM_2.5_ administrated cardiac tissue. However, dysfunction of mitochondria, especially that of the IFM, was enhanced in the myocardium of IR challenged PM_2.5_ treated animals that compromised the cardiac ability to recover from IR. In correspondence with the reduced cardiac hemodynamic indices in PM_2.5_ treated IR hearts, IR associated cardiac injury was significantly elevated in the PM_IR group, similar to the deteriorated mitochondrial functional changes in IR tissue. Furthermore, evaluating the in vitro mitochondrial protein translational capacity of IFM and SSM isolated from the different experimental groups suggested a noticeable deterioration in the in vitro protein translation capacity and the declined capacity was more prominent in IFM. Overall, in PM_2.5_ treated myocardium, we observed altered gene expression of mitochondrial bioenergetics, dynamics, mitophagy, and mitochondrial DNA maintenance proteins. This in turn adversely affects the ability of the heart to resist IR.

Mitochondria are abundant in cardiomyocytes and supply energy in the form of ATP to regulate cardiac contractility apart from its involvement in Ca^2+^ homeostasis [28]. About 90% of the cellular ATP is utilized to support the contraction–relaxation cycle within the myocardium, thereby ensuring that the cardiac energy demands are predominantly met by the mitochondria [29], especially the one present in myofibrils [30]. Previous studies have shown that when the energy demand of cardiac tissue was more elevated than the supply, which happens during ischemia followed by reperfusion, the dysfunction of mitochondria occurs due to calcium overload and the ROS attack [30]. Similar to the early findings, the present study demonstrated severe dysfunction of the IFM fraction of mitochondria in IR challenged myocardium. This finding was in agreement with the early reports that showed a higher cardiac injury and deterioration of mitochondria with co-existence of cardiac risk factors such as diabetic cardiomyopathy [31]. In the present study, PM_2.5_ administration prior to global ischemia and reperfusion is considered to be a co-existing risk factor prior to IR injury. Heavy meatal toxicity is reported to be a limiting factor for deteriorated cardiac function and its inefficient response to further oxidative stress [32]. 

PM_2.5_ from DPM contains different metal ions such as Ca, Mg, Al, S, K, Fe, Zn, Cu, Cr, Pb, Ba, and Ni [33] that can alter the cardiac physiology, induce changes in the cardiac ultrastructure, can inappropriately activate the proteolytic enzymes, promote oxidative stress by acting as a catalyst, and can also induce damage to mitochondria by getting deposited in the organelle. Accordingly, in the present study, we observed cardiac injury with PM 2.5, similar to the results reported by Bai and group [34], and also noted the increased oxidative stress [35]. Early reports indicate that iron can act as a catalyst in Fenton’s reaction and promote the free radical production [36], where the PM_2.5_ from DPM contains not only iron but also copper that can act as a catalyst for ROS production.

Early studies have shown that high lipid content and specific nature of basal membrane potential of mitochondria facilitate the transport of metals such as lead, cadmium, mercury, and manganese. In fact, the PM_2.5_ from DPM used in the present study contains lead which can modulate the activity of PGC1-a, the master regulator of bioenergetics function [37,38]. 

Maintaining the cellular mitochondrial health is mediated via different processes such as mitochondrial fission, fusion, biogenesis, and mitophagy [39]. Early studies have shown that an imbalance in the cellular energy demand due to environmental stress can alter the different mitochondrial processes and thus can adversely affect the overall mitochondrial quality [40].

Mitochondria are the metabolic hub of the cell and are involved in the regulation of different metabolisms linked to energy production, adaptive response, and cellular homeostasis [41]. Recently, investigators have identified more than one type of mitochondria that mediates the plethora of mitochondrial functioning in the heart. These mitochondria were initially identified based on their spatial location and are now identified in subpopulations that even differ in their function, morphology, and composition [42]. In order to meet the higher local energy demand supporting the contractile machinery in the myofibrils, IFM fraction of the mitochondria provides a constant supply of ATP [11]. Similarly, studies have shown that SSM fraction of mitochondria present near sarcoplasmic reticulum are mainly involved in regulating the production of different metabolites via acting as the site for metabolism or as the converging centre for several signaling molecules.

Early studies have shown that among the mitochondrial subpopulations, IR induced dysfunction is higher in the SSM than IFM. However, few studies with diseased hearts subjected to IR reported higher damage in the IFM fraction which in turn adversely affected cardiac contractility. The intact mitochondrial sarcoplasmic Ca^2+^ handling is a prerequisite for normal cardiomyocyte contraction. Accordingly, in the present study, the risk factor PrM_2.5_ induced severe dysfunction of IFM fraction with PM_2.5_ administration. Moreover, the ICPMS data in the present study showed an excessive Ca^2+^ in mitochondria of PM_2.5_ treated heart, which suggests a disruption of Ca^2+^ handling balance between mitochondria and sarcoplasmic reticulum. Being present in the myofibrils, IFM may be more sensitive towards the excessive Ca^2+^ from PM_2.5_ and that in turn deteriorates the functional efficiency of the contractile unit.

In addition, we noted low mitochondrial functional activity, especially in the IFM fraction where the ATP level was low, along with reduced ETC enzyme activity in PM_2.5_ treated cardiac tissues. Mitochondria, being a semi-autonomous organelle having their own molecular machinery and can independently replicate, translate, and transcribe mitochondrial DNA, the overall health of the organelle needs to be evaluated by considering all these events. However, ischemia reperfusion is an energy-dependent condition where the bioenergetic function of the mitochondria plays a critical role, which in turn are influenced by the impact of other mitochondrial events. In this direction, we identified that apart from the declined bioenergetic function, the impact of PM_2.5_ on other mitochondrial events such as fission and fusion helps in balancing the energy demand and supply.

PM_2.5_ induced mitochondrial toxicity was further confirmed at the subpopulation level by studying the in vitro protein translation capacity of both IFM and SSM isolated from different experimental groups. The in vitro protein translation capacity was significantly low in mitochondria obtained from PM_2.5_ administrated rat hearts. This decline was prominent in the IFM fraction, emphasizing the relevance of PM_2.5_ toxicity especially on the IFM. However, the selectivity of PM_2.5_ on IFM over SSM still lacks clarity and requires more studies at the basal level of mitochondrial subpopulations.

## 5. Conclusions

Administration of PM_2.5_ elevated the cardiac injury via compromising the physiological functioning of the heart via altering the hemodynamics. Additionally, we noted prominent mitochondrial bioenergetics functional decline, elevated oxidative stress, reduction in copy number, and modifications in mitochondrial fission, fusion, and mitophagy gene expression. These changes were more prominent in the IFM fraction of the mitochondria, which was further compromised in the presence of IR. Based on the above observations, we conclude that PM_2.5_ administration to the heart promotes the accumulation of metal ions from DPM in the mitochondria that in turn adversely affect the cardiac physiological function. The consequential impact of PM_2.5_ linked IFM damage was manifested in the deteriorated cardiac contractility and thus targeting specifically to the preservation of IFM may render cardio protection.

## Figures and Tables

**Table 1:** Primer details.

	Gene	Forward Primer	Reverse Primer
1	PGC-1α	5′-GAGGGACGAATACCGCAGAG-3′	5′-CTCTCAGTTCTGTCCGCGTT-3′
2	Dnm1	5′-TTGCCCTCTTCAACACTGAGC-3′	5′-ATGAAGCTGTCAGAGCCGTT-3′
3	Parkin	5′-AGTTTGTCCACGACGCTCAA-3′	5′-CAGAAAACGAACCCACAGCC-3′
4	MFN1	5′-TGACTTGGACTACTCGTGCG-3′	5′-GGCACAGTCGAGCAAAAGTG-3′
5	MFN2	5′-CTCTGTGCTGGTTGACGAGT-3′	5′-TCGAGGGACCAGCATGTCTA-3′
6	DRP1	5′-TGGAAAGAGCTCAGTGCTGG-3′	5′-TCAACTCCATTTTCTTCTCCTGT-3′
7	MFF	5′-GAAAACACCTCCACGTGTGC-3′	5′-CTGCTCGGATCTCTTCGCTT-3′
8	FIS	5′-CCAGAGATGAAGCTGCAAGGA-3′	5′-TTCCTTGAGCCGGTAGTTGC-3′
9	PINK1	5′-TGTATGAAGCCACCATGCCC-3′	5′-TCTGCTCCCTTTGAGACGAC-3′
10	TFAM	5′-GTTGCTGTCGCTTGTGAGTG-3′	5′-GTCTTTGAGTCCCCCATCCC-3′
11	β-actin	5′-GTGTGGTCAGCCCTGTAGTT-3′	5′-CCTAGAAGCATTTGCGGTGC-3′
12	POLG1	5′-CTTTGGGCTCCAGCTTGACT-3′	5′-TGGAGAAAATGCTTGGCACG-3′
13	Casp9-F	5’-GAGGATATTCAGCGGGCAGG-3’	5’-GCAGGAGATGAAGCGAGGAA-3’
14	Casp3-F	5’-CGGACCTGTGGACCTGAAAA-3’	5’-TAACCGGGTGCGGTAGAGTA-3’
15	Casp7-F	5’-TTCGACGGAAGACGGAGTTG-3’	5’-CCGGACATCCATACCTGTCG-3’
16	PARP-F	5’-ACCACGCACAATGCCTATGA-3’	5’-AGCAGTCTCCGGTTGTGAAG-3’
17	Glutaredoxin1-F	5’-AGCATGGCTCAGGACTTTGT-3’	5’-TTGAATCGCATTGGTGTTGT-3’
18	Peroxiredoxin 6-F	5’-CCTGGAGCAAGGACATCAAT-3’	5’-GTTTCTTGTCAGGGCCAAAA-3’
19	Peroxiredoxin 3- F	5’-CCAGAGTCCCCTACGATCAA-3’	5’-TGGCCACCTTTAACCTGAAC-3’
20	GUF 1	5’-GGTGTCTTGGAGCCAGGG-3’	5’-CCTCTTTCTCGCTCCACTTGT-3’
21	LRPPRC	5’-CAACAACATTGCTCTGGCCC-3’	5’-TCCACCTTGCCTGAATCCAC-3’
22	SLC25A4	5’-GGATCTTCCCAGCGTGAGTT-3’	5’-TGCACATTCTTGGGGTCTGG-3’

**Table 2:** ICPMS data showing metal deposition in mitochondria (values in PPM) (BDL: below detection limit) (*n* = 3 per group).

Metals	Normal	PM_C
Li	BDL	BDL
Be	BDL	BDL
B	BDL	0.01
Na	0.03	0.06
Mg	BDL	BDL
Al	BDL	BDL
P	BDL	BDL
K	0.04	0.06
Ca	0.01	0.02
Cr	BDL	BDL
Mn	0.03	0.04
Fe	0.05	0.09
Co	BDL	BDL
Ni	BDL	BDL
Cu	0.02	0.03
Zn	0.01	0.01
As	BDL	BDL
Cd	BDL	BDL
Pb	BDL	0.01

## Data Availability

The datasets generated during and/or analysed during the current study are available from the corresponding author on reasonable request.

## Supplementary Materials

The following supporting information can be downloaded at: https://www.mdpi.com/article/10.3390/biology11121811/s1, Supplementary File S1: Protein translation ability of mitochondria isolated from the experimental groups. Lanes: 1: Negative control, 2: Positive control, 3–6: IFM fraction of N, IR, PM_C, and PM_IR groups, 7–10: SSM fraction of N, IR, PM_C, and PM_IR groups.

## Abbreviations

PM: particulate matter; DPM: diesel particulate matter; IR: ischemia reperfusion injury; SSM: subsarcolemmal mitochondria; IFM: interfibrillar mitochondria; ROS: reactive oxygen species; ETC: electron transport chain; KH: Krebs–Henseleit buffer; OXPHOS: oxidative phosphorylation.

## References

[1] Lee B.J., Kim B., Lee K. (2014). Air pollution exposure and cardiovascular disease. Toxicol. Res..

[2] Waidyatillake N.T., Campbell P.T., Vicendese D., Dharmage S.C., Curto A., Stevenson M. (2021). Particulate Matter and Premature Mortality: A Bayesian Meta-Analysis. Int. J. Environ. Res. Public Health.

[3] Pardo M., Qiu X., Zimmermann R., Rudich Y. (2020). Particulate Matter Toxicity Is Nrf2 and Mitochondria Dependent: The Roles of Metals and Polycyclic Aromatic Hydrocarbons. Chem. Res. Toxicol..

[4] Liang S., Zhang J., Ning R., Du Z., Liu J., Batibawa J.W., Duan J., Sun Z. (2020). The critical role of endothelial function in fine particulate matter-induced atherosclerosis. Part. Fibre Toxicol..

[5] Zhang S., Lu W., Wei Z., Zhang H. (2021). Air Pollution and Cardiac Arrhythmias: From Epidemiological and Clinical Evidences to Cellular Electrophysiological Mechanisms. Front. Cardiovasc. Med..

[6] Tahrir F.G., Langford D., Amini S., Mohseni Ahooyi T., Khalili K. (2019). Mitochondrial quality control in cardiac cells: Mechanisms and role in cardiac cell injury and disease. J. Cell. Physiol..

[7] Sivakumar B., Kurian G.A. (2022). Diesel particulate matter exposure deteriorates cardiovascular health and increases the sensitivity of rat heart towards ischemia reperfusion injury via suppressing mitochondrial bioenergetics function. Chem. Biol. Interact..

[8] Sivakumar B., Kurian G.A. (2022). Inhalation of PM2.5 from diesel exhaust promote impairment of mitochondrial bioenergetics and dysregulate mitochondrial quality in rat heart: Implications in isoproterenol-induced myocardial infarction model. Inhal. Toxicol..

[9] Kuznetsov A.V., Javadov S., Margreiter R., Grimm M., Hagenbuchner J., Ausserlechner M.J. (2019). The Role of Mitochondria in the Mechanisms of Cardiac Ischemia-Reperfusion Injury. Antioxidants.

[10] Lu X., Thai P.N., Lu S., Pu J., Bers D.M. (2019). Intrafibrillar and perinuclear mitochondrial heterogeneity in adult cardiac myocytes. J. Mol. Cell. Cardiol..

[11] Hollander J.M., Thapa D., Shepherd D.L. (2014). Physiological and structural differences in spatially distinct subpopulations of cardiac mitochondria: Influence of cardiac pathologies. Am. J. Physiol. Heart Circ. Physiol..

[12] Hamanaka R.B., Mutlu G.M. (2018). Particulate Matter Air Pollution: Effects on the Cardiovascular System. Front. Endocrinol..

[13] Jiang Q., Ji A., Li D., Shi L., Gao M., Lv N., Zhang Y., Zhang R., Chen R., Chen W. (2021). Mitochondria damage in ambient particulate matter induced cardiotoxicity: Roles of PPAR alpha/PGC-1 alpha signaling. Environ. Pollut..

[14] Magnani N.D., Marchini T., Calabró V., Alvarez S., Evelson P. (2020). Role of Mitochondria in the Redox Signaling Network and Its Outcomes in High Impact Inflammatory Syndromes. Front. Endocrinol..

[15] Huang J., Li R., Wang C. (2021). The Role of Mitochondrial Quality Control in Cardiac Ischemia/Reperfusion Injury. Oxidative Med. Cell. Longev..

[16] Sivakumar B., Kurian G.A. (2022). PM2.5 Exposure Lowers Mitochondrial Endurance During Cardiac Recovery in a Rat Model of Myocardial Infarction. Cardiovasc. Toxicol..

[17] Holmuhamedov E.L., Oberlin A., Short K., Terzic A., Jahangir A. (2012). Cardiac subsarcolemmal and interfibrillar mitochondria display distinct responsiveness to protection by diazoxide. PLoS ONE.

[18] Shanmugam K., Ravindran S., Kurian G.A., Rajesh M. (2018). Fisetin Confers Cardioprotection against Myocardial Ischemia Reperfusion Injury by Suppressing Mitochondrial Oxidative Stress and Mitochondrial Dysfunction and Inhibiting Glycogen Synthase Kinase 3β Activity. Oxidative Med. Cell. Longev..

[19] Li J., Li H., Li H., Guo W., An Z., Zeng X., Li W., Li H., Song J., Wu W. (2019). Amelioration of PM2.5-induced lung toxicity in rats by nutritional supplementation with fish oil and Vitamin E. Respir. Res..

[20] Phillips J.E. (2017). Inhaled efficacious dose translation from rodent to human: A retrospective analysis of clinical standards for respiratory diseases. Pharmacol. Ther..

[21] Selley L., Schuster L., Marbach H., Forsthuber T., Forbes B., Gant T.W., Sandström T., Camiña N., Athersuch T.J., Mudway I. (2020). Brake dust exposure exacerbates inflammation and transiently compromises phagocytosis in macrophages. Met. Integr. Biometal Sci..

[22] Palmer J.W., Tandler B., Hoppel C.L. (1977). Biochemical properties of subsarcolemmal and interfibrillar mitochondria isolated from rat cardiac muscle. J. Biol. Chem..

[23] Ashar F.N., Moes A., Moore A.Z., Grove M.L., Chaves P., Coresh J., Newman A.B., Matteini A.M., Bandeen-Roche K., Boerwinkle E. (2015). Association of mitochondrial DNA levels with frailty and all-cause mortality. J. Mol. Med..

[24] Fernández-Silva P., Acín-Pérez R., Fernández-Vizarra E., Pérez-Martos A., Enriquez J.A. (2007). In Vivo and in organello analyses of mitochondrial translation. Methods Cell Biol..

[25] Khosravipour M., Safari-Faramani R., Rajati F., Omidi F. (2022). The long-term effect of exposure to respirable particulate matter on the incidence of myocardial infarction: A systematic review and meta-analysis study. Environ. Sci. Pollut. Res. Int..

[26] Robertson S., Thomson A.L., Carter R., Stott H.R., Shaw C.A., Hadoke P.W., Newby D.E., Miller M.R., Gray G.A. (2014). Pulmonary diesel particulate increases susceptibility to myocardial ischemia/reperfusion injury via activation of sensory TRPV1 and β1 adrenoreceptors. Part. Fibre Toxicol..

[27] Tong F., Zhang H. (2018). Pulmonary Exposure to Particulate Matter (PM2.5) Affects the Sensitivity to Myocardial Ischemia/Reperfusion Injury Through Farnesoid-X-Receptor-Induced Autophagy. Cell. Physiol. Biochem..

[28] Lukyanenko V., Chikando A., Lederer W.J. (2009). Mitochondria in cardiomyocyte Ca2+ signaling. Int. J. Biochem. Cell Biol..

[29] Brown D.A., Perry J.B., Allen M.E., Sabbah H.N., Stauffer B.L., Shaikh S.R., Cleland J.G., Colucci W.S., Butler J., Voors A.A. (2017). Expert consensus document: Mitochondrial function as a therapeutic target in heart failure. Nat. Rev. Cardiol..

[30] Kurian G.A., Rajagopal R., Vedantham S., Rajesh M. (2016). The Role of Oxidative Stress in Myocardial Ischemia and Reperfusion Injury and Remodeling: Revisited. Oxidative Med. Cell. Longev..

[31] Mahalakshmi A., Kurian G.A. (2018). Evaluating the impact of diabetes and diabetic cardiomyopathy rat heart on the outcome of ischemia-reperfusion associated oxidative stress. Free. Radic. Biol. Med..

[32] Alissa E.M., Ferns G.A. (2011). Heavy metal poisoning and cardiovascular disease. J. Toxicol..

[33] Farahani V.J., Pirhadi M., Sioutas C. (2021). Are standardized diesel exhaust particles (DEP) representative of ambient particles in air pollution toxicological studies?. Sci. Total Environ..

[34] Bai L., Zhao Y., Zhao L., Zhang M., Cai Z., Yung K., Dong C., Li R. (2021). Ambient air PM_2.5_ exposure induces heart injury and cardiac hypertrophy in rats through regulation of miR-208a/b, α/β-MHC, and GATA4. Environ. Toxicol. Pharmacol..

[35] Vincent R., Kumarathasan P., Goegan P., Bjarnason S.G., Guénette J., Bérubé D., Adamson I.Y., Desjardins S., Burnett R.T., Miller F.J. (2001). Inhalation Toxicology of Urban Ambient Particulate Matter: Acute Cardiovascular Effects in Rats.

[36] Fischbacher A., von Sonntag C., Schmidt T.C. (2017). Hydroxyl radical yields in the Fenton process under various pH, ligand concentrations and hydrogen peroxide/Fe(II) ratios. Chemosphere.

[37] Meyer J.N., Leung M.C., Rooney J.P., Sendoel A., Hengartner M.O., Kisby G.E., Bess A.S. (2013). Mitochondria as a target of environmental toxicants. Toxicol. Sci..

[38] Tinkov A.A., Nguyen T.T., Santamaria A., Bowman A.B., Buha Djordjevic A., Paoliello M., Skalny A.V., Aschner M. (2021). Sirtuins as molecular targets, mediators, and protective agents in metal-induced toxicity. Arch. Toxicol..

[39] Youle R.J., van der Bliek A.M. (2012). Mitochondrial fission, fusion, and stress. Science.

[40] Duarte-Hospital C., Tête A., Brial F., Benoit L., Koual M., Tomkiewicz C., Kim M.J., Blanc E.B., Coumoul X., Bortoli S. (2021). Mitochondrial Dysfunction as a Hallmark of Environmental Injury. Cells.

[41] Weinberg S.E., Sena L.A., Chandel N.S. (2015). Mitochondria in the regulation of innate and adaptive immunity. Immunity.

[42] Kuznetsov A.V., Margreiter R. (2009). Heterogeneity of mitochondria and mitochondrial function within cells as another level of mitochondrial complexity. Int. J. Mol. Sci..

