# Supplementary material for: Consequential Impact of Particulate Matter Linked Inter-Fibrillar Mitochondrial Dysfunction in Rat Myocardium Subjected to Ischemia Reperfusion Injury
Source: Biology (Basel). 2022 Dec 13;11(12):1811. doi: 10.3390/biology11121811 (PMC9775305; doi:10.3390/biology11121811)
Supplement: Supplementary file 1 [file biology-11-01811-s001.zip › biology-2044589-supplementary.pdf]

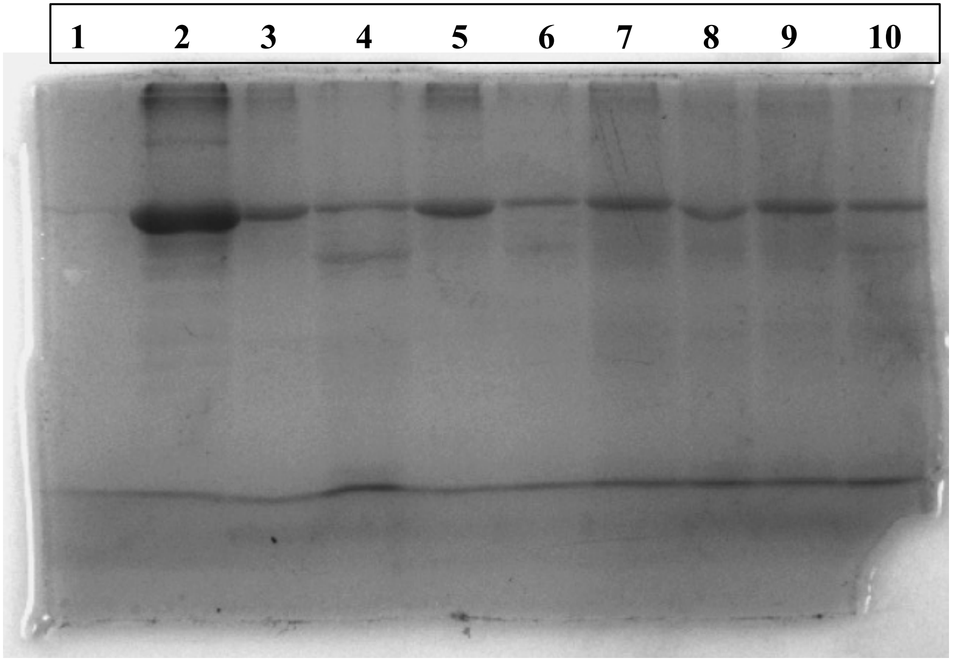

Gel image for figure 8: Lanes: 1: Negative control, 2: Positive control, 3-6: IFM fraction of N, IR, PM_C and PM_IR groups, 7-10: SSM fraction of N, IR, PM_C and PM_IR groups.
